# Dietary energy density and adiposity: Employing bias adjustments in a meta-analysis of prospective studies

**DOI:** 10.1186/1471-2458-11-48

**Published:** 2011-01-22

**Authors:** Désirée C Wilks, Adrian P Mander, Susan A Jebb, Simon G Thompson, Stephen J Sharp, Rebecca M Turner, Anna Karin Lindroos

**Affiliations:** 1 Medical Research Council Human Nutrition Research, Cambridge, UK; 2 Medical Research Council Biostatistics Unit, Cambridge, UK; 3 Medical Research Council Epidemiology Unit, Cambridge, UK

## Abstract

**Background:**

Dietary studies differ in design and quality making it difficult to compare results. This study quantifies the prospective association between dietary energy density (DED) and adiposity in children using a meta-analysis method that adjusts for differences in design and quality through eliciting and incorporating expert opinion on the biases and their uncertainty.

**Method:**

Six prospective studies identified by a previous systematic literature search were included. Differences in study quality and design were considered respectively as internal and external biases and captured in bias checklists. Study results were converted to correlation coefficients; biases were considered either additive or proportional on this scale. The extent and uncertainty of the internal and external biases in each study were elicited in a formal process by five quantitatively-trained assessors and five subject-matter specialists. Biases for each study were combined across assessors using median pooling and results combined across studies by random-effects meta-analysis.

**Results:**

The unadjusted combined correlation between DED and adiposity change was 0.06 (95%CI 0.01, 0.11; p = 0.013), but with considerable heterogeneity (I^2 ^= 52%). After bias-adjustment the pooled correlation was 0.17 (95%CI - 0.11, 0.45; p = 0.24), and the studies were apparently compatible (I^2 ^= 0%).

**Conclusions:**

This method allowed quantitative synthesis of the prospective association between DED and adiposity change in children, which is important for the development of evidence-informed policy. Bias adjustment increased the magnitude of the positive association but the widening confidence interval reflects the uncertainty of the assessed biases and implies that higher quality studies are required.

## Background

The prevalence of obesity in childhood is increasing around the world and is causally linked to large predicted increases in morbidity [1-4]. The fundamental physiological cause of weight gain is a positive energy imbalance, generally caused by excessive energy intake [2]. However, the dietary components that contribute to excess energy intake are not clear, which hampers policy making to prevent obesity. Research suggests that dietary energy density (DED, food energy/food weight) is an important determinant of total energy intake and experimental studies have shown that a low DED leads to a lower ad libitum energy intake in both adults and children [5-7]. The evidence from observational studies, however, is sparse. A recent narrative systematic review found that most studies reported positive prospective associations between DED and adiposity in adults and children [8] though the studies varied considerably in their design and quality such that it was not possible to produce an overall pooled estimate of the association. For example studies include either adults or children and follow-up times range between 9 months and 7 years. In addition, there is diversity in the way DED is modelled in the statistical analysis (continuous vs. categorical), the calculation of DED (including food only, food and all drinks, or food and caloric drinks), and the measures used for obesity status (e.g. body weight or fat mass) [8].

To overcome this diversity problem and to be able to estimate an overall pooled estimate of the association, we have adapted and applied a recently developed experimental meta-analysis method that allows adjustment for differences in study design and quality through a formal process of eliciting and incorporating expert opinion [9]. This method attempts to quantify the biases and their uncertainty, independently of the results, rather than to ignore them and produce a pooled association which is difficult to interpret. Although use of expert opinion may be considered controversial, meta-analysts routinely rely on even stronger judgements when excluding some studies altogether and regarding those included as unbiased. Moreover, policy makers faced with imperfect evidence use expert opinion informally in making judgements and decisions. The aim of this research was to formalise this process, making it transparent and accountable, and use this novel meta-analysis method to quantitatively synthesize the evidence on the prospective association between DED and change in fat mass index (FMI, fat mass/height^2^) in children.

## Methods

### Source studies

This meta-analysis considered all cohort studies investigating the prospective associations between DED and measures of adiposity (n = 8) [10-17], which are presented in a previous systematic review [8]. Studies included in the review [8] were English language publications before 1 September 2008 and were either longitudinal or cross-sectional observational studies that investigated the associations between DED and measures of body weight and composition in free-living adults and children, excluding those currently actively participating in weight loss interventions or samples limited to clinically ill participants. Only published studies were considered and we did not contact authors for further details. For this meta-analysis we further excluded studies that do not present a truly prospective analysis [11] or originate from trials including intentional weight loss in the past [10]. Six eligible studies thus remained [12-17] (summarized in Table 1). Studies in children or adults were considered potentially relevant to our research question and our analysis adjusts for differences in study design that are expected to affect the observed association.

**Table 1:** Summary of study characteristics

Study	Population	Follow-up	Method; Exposure	Outcome	Confounding
Butte et al [12]	1030 4 - 19y old Hispanic children/adolescences, who are either overweight or have ≥ 1 overweight sibling, USA.	1y	24 hr recall; DED_FC_	BW gain (kg/y)	Sex, age, age^2^, Tanner stage and BMI, all assessed at *BL*.
Deierlein et al [13]	2006 16-47y old pregnant women (≥ 16y), USA.	~9mo	FFQ; DED_FC_: Quartile 1-4 = 0.71, 0.86, 0.98 and 1.21	Gestational BW gain	Pregravid BMI, gestational age and residual energy intake.
Iqbal et al [14]	2025 30-60y old male and female adults. The 1936 cohort and WHO MONICA1, Dk.	5y	Diary; DED_FD_	Change in BW	BMI, age, leisure time physical activity, smoking status, educational level all assessed at *BL; *cohort.
Johnson et al [15]	1432 7y old children; ALSPAC, UK.	2y	Diary; DED_FO_	Adiposity defined by FMI; Quintile 5 vs. 1 - 4	*BL *energy misreporting, total EI, EI from drinks, dietary fat and fiber intake, sex, *BL *overweight, TV watching, maternal BMI and education.
McCaffrey et al [16]	115 6-8y old children from Coleraine, Northern Ireland.	7y	Diary; DED_FO_	FMI; Tertile 3 vs. 1 and 2	Sex, *BL *diet misreporting and *FU *Tanner stage.
Savage et al [17]	192 24-47y old Non-Hispanic women (*n*: Tertiles 1-3 = 61, 63&59) living in Pennsylvania, USA.	6y	24 hr recall; DED_FO_: Tertiles 1-3 = 1.3, 1.7 and 2.1	Change in BW	The analysis of interest was unadjusted.

BW = body weight; *BL *= baseline; BMI = body mass index; EI = energy intake; FFQ = food frequency questionnaire; DED_FC _= DED of food and caloric drinks; DED_FD _= DED of food and drinks; DED_FO _= DED of food only; h = hour; *FU *= follow-up; *n *= sample size.

### Application of the bias-adjustment method

The bias-adjustment method is described in detail by Turner et al. [9]. The steps used to implement this method for the six studies included in this meta-analysis are outlined below. The study by Savage et al. [17] is used throughout the paper to exemplify the method.

### Target question and target setting

The precise definition of the public health target question, which the meta-analysis aims to address, was agreed as "Is dietary energy density associated with change in fat mass index in children?"

The target setting, which describes the optimal study protocol answering the target question with regards to study population, exposure and outcome measures and follow-up time, was defined as:

(i) General population of children aged 4-11yrs in the UK

(ii) DED assessed by 7 day weighed food diaries calculated by dividing total food energy (kJ) by total food weight (g) excluding beverages

(iii) Change in FMI (fat mass/height^2^, kg/m^2^), measured from baseline to follow-up

(iv) Outcome assessed 2 years after the baseline measurement.

The target setting focuses on children between 4 and 11 years excluding both baby to toddler stages and advanced stages of puberty to match the policy focus on the UK Healthy Weight, Healthy Lives strategy [18]. DED calculated as the ratio of total food energy to total food weight was used as the target exposure because inclusion of beverages disproportionately influences DED and water intake is often reported inaccurately [8]. FMI was used as the measure of adiposity in children due to its independence of growth rates [19]. Two year follow-up was selected since we anticipated diminished associations with increasing follow-up time, whereas shorter follow-up times would not adequately allow for the slow accumulation of fat mass.

### Idealised studies

For each study included in the meta-analysis, an idealized version was defined. The idealized study is an imagined repeat of the actual study with modifications to eliminate all sources of internal biases such as selection bias, attrition bias, inappropriate adjustment for confounding and biases arising from how the exposure and outcome were measured. The design of the idealized study does not need to be practically feasible. For example, the idealized study of Savage et al. [17] (Table 1) included the following elements:

(i) Non-Hispanic women living in central Pennsylvania, USA

(ii) DED assessed by three 24 h recalls, including two weekdays and one weekend day and calculated by dividing total food energy by total food weight

(iii) Change in body weight measured from baseline to follow-up

(iv) Outcome assessed six years after the baseline measurement.

### Internal and external biases

Potential internal biases in each study were identified by comparing the study against its idealized version. For this meta-analysis, internal biases were categorized as biases related to the measurement of the outcome ("outcome bias") and the exposure ("exposure bias"), missing data and loss to follow-up ("attrition bias"), appropriate confounders ("confounding bias") and whether the inclusion and exclusion criteria were clear and adhered to ("selection bias"). Biases related to inappropriate statistical analysis or any other flaws were included in a separate category ("other bias suspected"). Important variables potentially related to both the outcome and exposure were considered by the subject matter specialists and statisticians and the following reference set of confounders was selected: energy-containing beverages, sex, total energy intake or energy misreporting, socio-economic status, ethnicity, some measure of baseline body size, physical activity and, depending on the age of the study population, Tanner stage and smoking status. Many DED studies adjust for fat and fiber intake; we did not consider these as confounders as both are expected to be direct determinants of DED. The adjustment for confounding in each study was judged against this reference set.

Potential external biases were identified by comparing each idealized study against the target setting. External biases were categorized as biases related to the follow-up time ("timing bias"), the presented outcome ("outcome bias") and exposure measures ("exposure bias") and the study population ("population bias").

Bias checklists were prepared for each study, highlighting information that might be relevant in the assessment of each of the possible internal and external biases. To ensure consistency, biases were identified by the same subject-matter specialist together with one statistician for each study.

### Common scale for study results

The studies expressed the association between baseline DED and change in adiposity differently, with a mix of regression coefficients, summary statistics, odds ratios and p-values. To allow pooling of results it was necessary to transform the associations onto a common metric. From the available information a p-value could be calculated for the association in each study and, using the respective sample size, this was subsequently transformed into a correlation and standard error for each study. Biases were assessed on the correlation scale. To do the calculations we applied a Fisher-transformation to the correlations, the Fisher-transformed correlation being *z *= 0.5ln[(1+*r*)/(1-*r*)] and *z *having a standard error SE(z) = 1/√ (*n*-3), where *r *is the correlation and *n *is the sample size. The number of SEs *z *is away from zero is derived from the *p*-value, thus providing an estimate of *z*. We back-transformed *z *to the correlation *r *in order to present results. The Fisher-transformed and original scales were in fact almost identical in the range - 0.3 to +0.3.

### Bias elicitation meetings

**Figure 1:**
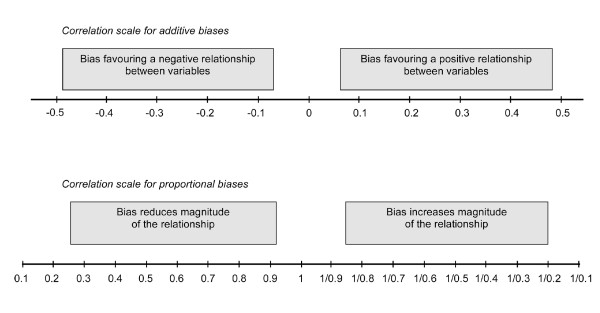
**Elicitation scales for quantifying additive and proportional biases**.

Internal bias elicitation meetings involved five quantitatively-trained assessors and separate external bias elicitation meetings involved five assessors with subject-matter knowledge. At the meetings, each study report and bias checklist were discussed in turn in a non-quantitative manner. The discussion included consideration of whether each bias would only change the magnitude of the association (a proportional bias) or if it could change the direction of the association (an additive bias). Following the discussion, each assessor independently provided their quantitative opinion on the impact and uncertainty of each of the biases on a bias elicitation form. The biases were indicated using 67% intervals, such that the assessor thought that the bias was twice as likely to lie inside the interval as outside it. The additive and proportional elicitation scales for quantifying internal and external biases are given in Figure 1. For example if the assessor believed that a study with 18 months follow-up instead of 2 years introduced a small proportional timescale bias then a possible 67% interval could be (0.9,1). If the assessor believed that a study of adults was not relevant to the target question in children, then a very wide interval would be indicated; the assessor would effectively give zero weight to adult studies in the pooled estimate.

### Incorporating the bias elicitations into the meta-analysis

The elicited internal biases from each assessor were used to calculate the mean and variance of the total additive and total proportional bias for each study, which were then used to adjust the estimated correlation coefficients and standard errors. The same process was used to adjust these results for the external biases. All calculations used formulae adapted from Turner et al [9]. The results were pooled across assessors, using the median estimate and the median standard deviation; such median pooling corresponds to a "typical" assessor [20]. Finally, the fully adjusted results were combined across studies using random-effects meta-analysis. Statistical heterogeneity was assessed using the I^2 ^statistic [21], which gives the percentage of variation between the study estimates attributable to true between-study heterogeneity rather than random variation; 0% indicates no heterogeneity. Analyses were conducted using STATA 11.0 (StataCorp 2009. College Station, TX: StataCorp LP).

## Results

### Study characteristics and extracted results

Table 1 summarizes the six eligible prospective studies on DED and change in adiposity. Three studies included either children [15,16] or adolescents [12]. The other three studies analyzed male and/or female adults up to 60 years [13,14,17], of which one investigated pregnant women [13]. Studies were carried out either in the USA [12,13,17] or in Europe [14-16]. Ethnicity was generally representative of the population from the place of study. Initial participant numbers per study ranged from 115 to 2025, while the analyses included between 48 and 1762 participants. Outcome was assessed after approximately 9 months to 7 years follow-up.

DED was assessed by food diaries [14-16], 24 h recalls [12,17] or a food frequency questionnaire [13]. In three of the six studies DED of food was reported and used as the exposure in the analyses [15-17]. Two studies reported DED of food and energy-containing beverages [12,13] and one study DED of food and all drinks [14]. In four studies change in body weight from baseline to follow-up was reported as the outcome [12-14,17], while two studies used the follow-up FMI [15,16]. With the exception of the study by Savage et al. [17] that presented unadjusted results, the estimated prospective association between DED and adiposity was adjusted for confounding factors. Depending on the study these included sex, age or Tanner stage, a baseline measure of body composition, energy intake and macronutrients or fiber intake, socioeconomic status, smoking and physical activity or inactivity.

Table 2 presents the results extracted from each study along with calculated correlation coefficients. Four studies reported positive prospective associations between baseline DED and change in adiposity, with correlation estimates between 0.06 and 0.33 [13,15-17]. Correlation estimates from the other two studies were close to zero [12,14].

**Table 2:** Correlation coefficients of studies calculated from *p*-values according to the principal results extracted

Study (source for extracted results)	Extracted results		Re-calculated results		
	
	N	Model & results	p-value	z (SE)	r
Butte et al [12]*(Table 2)*^a^	548	GEE: *β *= 0.23; SE = 0.35; p = 0.5		0.029 (0.043)	0.029
Deierlein et al [13]*(Table 2) *^b^	1231	Quartile mean differences from Q_1_: Q_2 _0.49; CI - 0.4,1.37; Q_3 _1.13; CI 0.24,2.01; Q_4 _1.08; CI 0.20,1.97	0.046	0.057 (0.029)	0.057
Iqbal et al [14]*(Table 4)*^c^	1762	LM;*β*- 13.49; SE = 36.46; p = 0.711		-0.009 (0.024)	-0.009
Johnson et al [15]*(Table *3, *Model 4)*	584	GLM; OR = 1.36; CI 1.09,1.69	0.006	0.114 (0.042)	0.114
McCaffrey et al [16]*(Table 6, Model 2)*	48	GLM; OR = 2.16; CI 1.099, 4.25; p = 0.026		0.332 (0.149)	0.320
Savage et al [17]*(Text)*^d^	183	Tertile means: T_1 _2.5, SD = 6.8; T_2 _4.8, SD = 9.2; T_3 _6.4, SD = 6.5	0.067	0.137 (0.075)	0.136

β = regression coefficient; CI = confidence interval; GEE = generalized estimated equations; GLM = generalized linear model; LM = linear regression model; *n *= sample size; OR = odds ratio; p = p-value; *r *= correlation; SD = standard deviation; SE = standard error; T = tertile; Q = quintile; *z *= Fisher transformed correlation.

^a^The p-value was derived from the GEE regression coefficient. The GEE model assumed an exchangeable correlation structure within families. An intra-family correlation coefficient of c = 0.26 is reported. The design effect is 1.46 = 1+(m-1)*c = 1+(879/319-1)*0.26, where m is the average number of people within each family, and thus the effective sample size is 548 (= 796/1.46). It is assumed that the average cluster size of 879/319 is correct for the 798 individuals included in the analysis. ^b^The regression slope and p-value were obtained from a weighted regression using analytical weights in Stata. A complete balance across quartiles has been assumed and the mean differences in DED were calculated from the published table. The p-value and sample size were used to calculate the implied correlation.^c ^The paper reports results for males and females separately; groups have been merged. ^d^P-value was re-estimated using weighted regression.

### Bias-adjusted meta-analysis

Internal biases suspected to affect all source studies were selection, attrition and/or confounding biases. For example, insufficient information had been provided regarding the recruitment strategy in the source studies and related papers and it seemed likely that losses to follow-up or immediate dropouts had affected the results [12-17] (Table 3). Confounding bias was suspected mainly because not all relevant confounders were adjusted for or the choice of confounders was not justified. Moreover, there were some issues regarding exposure and outcome biases, for instance due to unknown misreporting of the diet or ambiguity regarding the measurement times.

**Table 3:** Important internal additive biases identified in the studies

Study	Selection bias	Attrition bias	Confounding bias
Butte et al [12]	□ No information about immediate drop-outs; □ Recruitment not random.	□ 51 drop-outs, 81 exclusions from the analysis; □ Unclear whether drop-outs and exclusions differ from completers	□ Inappropriate adjustment; □ No stated justification for using confounders; □ Tanner stage assessed by self-report.
Deierlein et al [13]	□ Selections of clinics unclear.	□ ~12% losses to *FU*; □ ~30% exclusions from the analysis, who differ from completers.	□ Inappropriate adjustment; □ Self-reported pregravid BW; □ Assessment time unclear.
Iqbal et al [14]	□ Few inclusion, exclusion criteria&details of the original study cohorts; □ *BL *measures missing for 13% of the participants, unclear if they differ from those included.	□ Participation rate of 79%; □ 3 exclusions from the analysis.	□ Inappropriate adjustment; □ No stated justification for using confounders; □ Assessment of only leisure time PA; □ Measurement of confounders unclear.
Johnson et al [15]		□ 52% of children with incomplete datasets (little difference to children with complete datasets).	□ Inappropriate adjustment; □ Self-statement of parental BW and height; □ Time point of assessment of TV watching habits unclear.
McCaffrey et al [16]	□ Little information on the recruitment strategy, inclusion and exclusion criteria.	□ 58% of children were lost to *FU *(little difference to completers); □ 2 children were excluded from the analysis.	□ Inappropriate adjustment; □ Tanner stage assessed by self-report.
Savage et al [17]	□ No data describing the study sample.	□ 88% retention rate; □ Of the 68 women, dietary data were missing for 3, 9&18 women at years 2, 4&6.	□ The extracted model is unadjusted.

BW = body weight; *BL *= baseline; *FU *= follow-up; PA = physical activity.

External biases were expected because of differences between the source studies and the pre-defined target setting regarding the way the diet was assessed and DED was calculated, the varying outcome measures, differences in the population as well as the follow-up time (Table 1).

**Figure 2:**
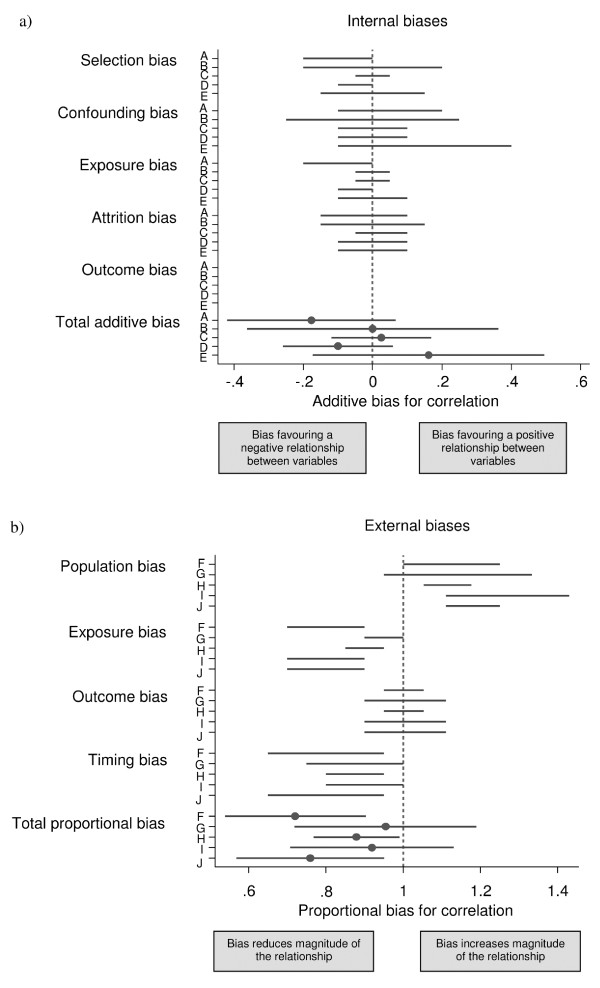
**Assessment of biases for the study by Savage et al**. Assessment of biases for the study by Savage et al [17]. In this study all internal biases were additive and all external biases were proportional. Internal biases were elicited from 5 assessors (A-E) and external biases from 5 assessors (F-J). Ranges indicate 67% intervals for the bias, so the bias is considered twice as likely to be inside the interval as outside it. A blank indicates no bias for that category.

Figure 2 shows the individual assessments of each bias assessor on the elicitation scales for (a) internal and (b) external biases in the study by Savage et al. [17]. In this study all internal biases were considered additive and all external biases proportional. Internal biases were generally distributed around zero, suggesting that the bias would be equally likely to favor a positive or negative association, although the uncertainty was large. The proportional external biases varied in size and assessors often expressed considerable uncertainty, while there was agreement on how the bias may have affected the correlation (reduced vs. increased magnitude). The combined external biases of each respective assessor were distributed around 0.85, indicating that the biases are believed to be likely to induce an attenuated association between exposure and outcome (Figure 2b). Despite the fact that biases were elicited independently, there was a general degree of consistency amongst assessors.

**Figure 3:**
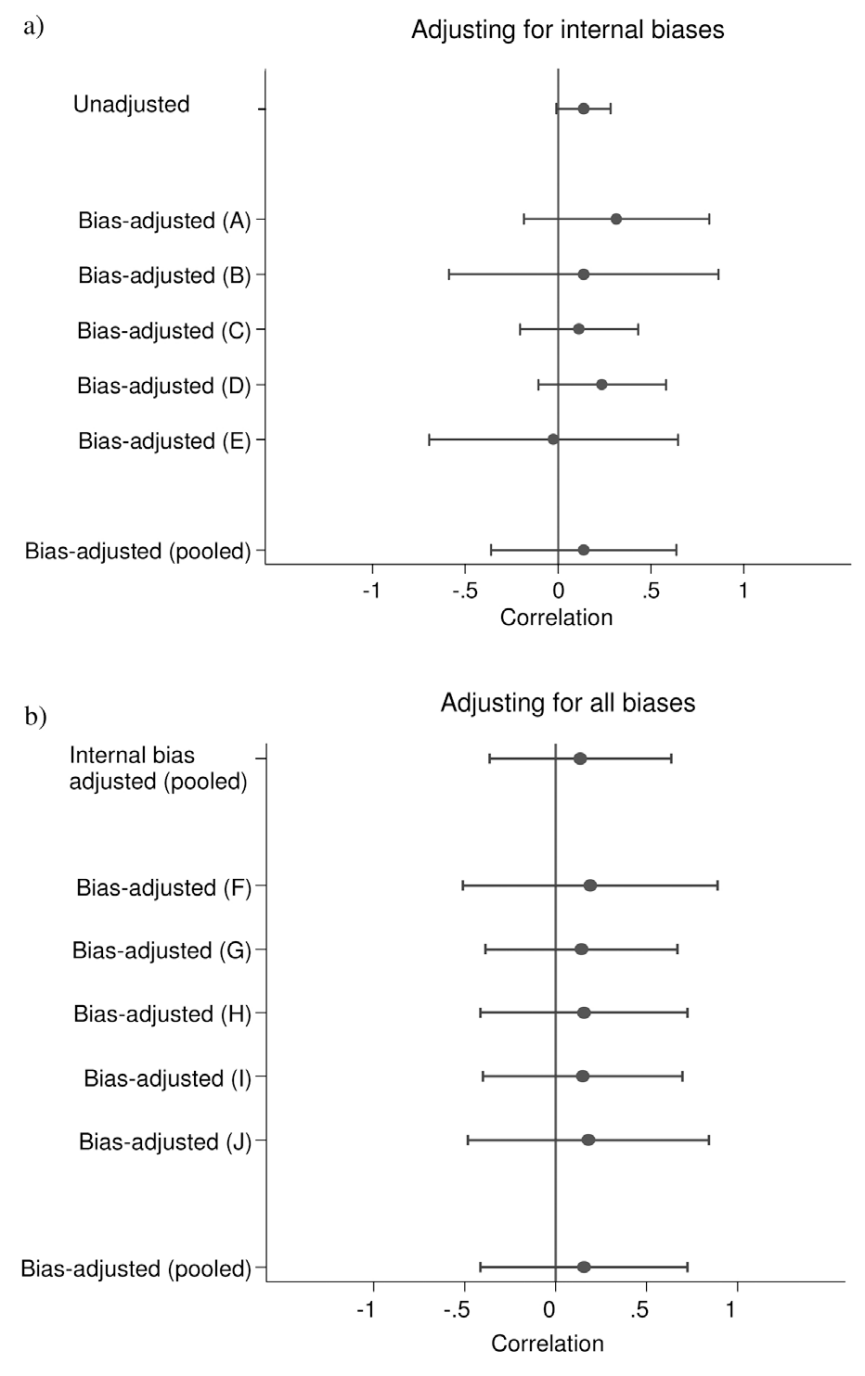
**Impact on correlations of adjusting for bias for the study by Savage et al**. Impact on correlations (95% CIs) of adjusting for bias for the study by Savage et al [17], for the assessors (A-E and F-J) separately and combined using median pooling. Values on the right hand side of the x-axis represent a positive correlation between dietary energy density and change in adiposity, i.e. greater baseline dietary energy density is related to a greater increase in adiposity.

The impact of adjusting the estimated correlation from the study by Savage et al. [17] for first internal and then external biases is illustrated in Figure 3. Neither adjustment for internal nor external biases affected the direction of the result considerably, although the 95% confidence intervals widened markedly, reflecting the assessors' uncertainty about the impact of the biases.

**Figure 4:**
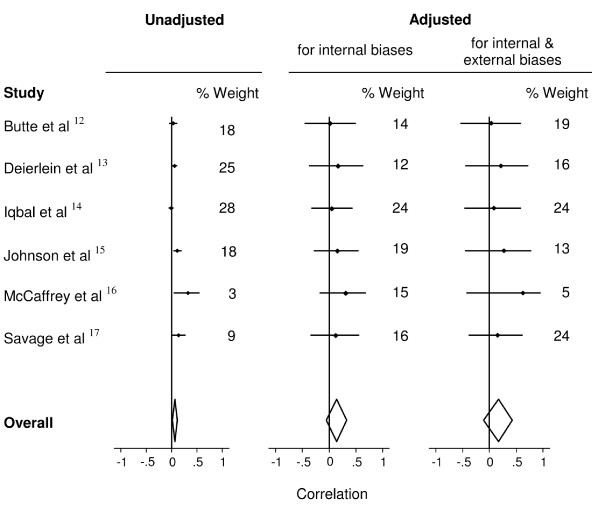
**Random-effects meta-analyses of the six studies**. Random-effects meta-analyses of six studies evaluating the prospective associations of dietary energy density and subsequent change in adiposity in children - (a) unadjusted, (b) adjusted for internal biases and (c) adjusted for internal and external biases. The correlation in each source study and the combined correlation are shown, along with 95% confidence intervals and % weight each study contributes to the overall result.

Figure 4 shows the estimated correlations between DED and change in adiposity (a) unadjusted, (b) adjusted for internal biases and (c) adjusted for both internal and external biases, for each study and combined across studies using random effects meta-analysis. The estimated correlation from the meta-analysis of the unadjusted study results was 0.06 (95%CI 0.01, 0.11; p = 0.013), but there was considerable heterogeneity (I^2 ^= 52%) making it difficult to interpret. After adjustment for internal biases that reflects lack in study quality, the pooled correlation was 0.14, with a widened confidence interval (95%CI - 0.06, 0.34; p = 0.16) and studies were apparently compatible (I^2 ^= 0%). After adjusting for both internal and external biases, which allows drawing conclusions that are specific to the particular target setting, the pooled correlation between DED and subsequent change in FMI in children was 0.17 (95%CI - 0.11, 0.45; p = 0.24) and the results remained compatible (I^2 ^= 0%).

## Discussion

This meta-analysis with bias-adjustment allows a quantitative evaluation of the totality of the evidence on the prospective relationship between DED and the change in FMI in children. A previous narrative review reported that decreasing the energy density of the diet may offset weight gain in childhood. However, because of the lack of prospective DED studies, their heterogeneous nature and the difficulties in comparing results presented in different ways, the evidence has not been synthesized quantitatively. This limits the possibility of drawing an overall conclusion and making policy related decisions.

Our analysis provides an overall quantitative synthesis of the evidence-base for decision-makers. The unadjusted results from the six studies gave a combined correlation of baseline DED with change in adiposity of 0.064 (95%CI 0.01, 0.11; p = 0.013). After bias adjustment the association between DED of food and the change in FMI in children for the target setting was 0.17 (95%CI - 0.11, 0.45; p = 0.24). Relative to the unadjusted analysis, the magnitude of the correlation coefficient was increased, indicating the possibility that DED is an important determinant of excess weight gain. However, the confidence interval widened after bias-adjustment, which is due to incorporating the assessors' uncertainty regarding the size of the biases, and implies that higher quality studies are required. The statistical heterogeneity among studies was large in the unadjusted meta-analysis (I^2 ^= 52%), which therefore limits interpretability. The bias-adjustment process eliminated the heterogeneity amongst studies (I^2 ^= 0%). Thus, while the association is no longer statistically significant, the data can now be interpreted with a clearer understanding of the biases. In our view, the magnitude of the correlation provides increased support to policymakers for interventions to reduce DED to prevent obesity in children, and for advice to consumers of the importance of reducing dietary energy density.

The process of bias-adjustment, at the heart of this method, relies on expert opinion and might be considered to be somewhat subjective. We do not claim that the elicited bias distributions are 'correct'; we are dealing with epistemic uncertainty, and they express judgements about our beliefs. However, the opinions of several experts are combined so that individual opinions do not unduly influence the final result of the meta-analysis. The experts were chosen for their quantitative or subject-matter skills, and we prefer to incorporate their judgements rather than simply ignore the suspected biases in the studies available. In addition, consistency across studies and transparency is ensured by the very structured and systematic process of bias-adjustment. Although some opinions on biases varied between the assessors, the differences were in general quite small (Figure 2) and mainly related to the width of intervals reflecting different levels of uncertainty about the effect of the biases. Hence, the adjusted estimates for individual assessors were similar to the pooled adjusted estimate (Figure 3).

A similar bias-adjusted meta-analysis has already been conducted for a systematic review of prospective observational studies of physical activity and subsequent gain in fat mass in children [22]. This method may also be more widely applicable for evidence synthesis across a range of other areas in the population health sciences where studies often cannot be pooled in conventional meta-analyses due to their heterogeneity and differences in design and quality.

## Conclusions

This bias-adjustment meta-analysis allowed quantitative synthesis of the prospective association between DED and change in adiposity in children. The result indicates that DED may be an important dietary determinant of unhealthy weight gain in children. Our analysis emphasizes the need for higher quality studies with more precise measurements of dietary intake and body composition and presentation of adequate analyses.

## Authors' contributions

This is a collaborative project between nutritionists, epidemiologists and statisticians from three UK Medical Research Council units. All authors contributed to the study design, the bias assessments, and drafting the paper. RMT, SGT and SAJ initiated the study, DCW carried out the systematic review and coordinated the expert groups, APM undertook the statistical analyses with input from SJS, and AKL coordinated the study. All authors have given final approval of the version to be published.

## Pre-publication history

The pre-publication history for this paper can be accessed here:

http://www.biomedcentral.com/1471-2458/11/48/prepub
